# Supplementary material for: Frequency and Spectrum of Mutations Induced by Gamma Rays Revealed by Phenotype Screening and Whole-Genome Re-Sequencing in Arabidopsis thaliana
Source: Int J Mol Sci. 2022 Jan 7;23(2):654. doi: 10.3390/ijms23020654 (PMC8775868; doi:10.3390/ijms23020654)
Supplement: Supplementary file 1 [file ijms-23-00654-s001.zip › ijms-1501962-supplementary/supplementary/Supplementary Table S1. Sequencing data generated and mapping to the Arabidopsis thaliana genome.pdf]

Table S1. Sequencing data generated and mapping to the *Arabidopsis thaliana* genome

Line	Number of Reads	Average coverage (×)	Mapping quality
G200	19553765	23.48	57.27
G240	21445199	19.09	57.24
G266	21154467	21.09	57.23
G287	16698866	16.14	57.24
G320	18783066	22.45	57.29
G431	21952290	25.41	57.30
G692	18518084	21.67	57.28
